# Circulating tumor DNA analysis: potential to revise adjuvant therapy for stage II colorectal cancer

**DOI:** 10.1038/s41392-022-01164-y

**Published:** 2022-09-05

**Authors:** Ning Zhu, Hanguang Hu, Ying Yuan

**Affiliations:** 1 Department of Medical Oncology, The Second Affiliated Hospital, Zhejiang University School of Medicine, Hangzhou, Zhejiang China; 2 Cancer Institute, Key Laboratory of Cancer Prevention and Intervention, Ministry of Education, The Second Affiliated Hospital, Zhejiang University School of Medicine, Hangzhou, Zhejiang China; 3 Cancer Center, Zhejiang University, Hangzhou, Zhejiang China

**Keywords:** Gastrointestinal cancer, Predictive markers, Cancer therapy

In the DYNAMIC multicenter phase 2 study recently published in The New England Journal of Medicine, Tie J. et al. demonstrated that a novel circulating tumor DNA (ctDNA)-guided regimen reduced the use of adjuvant chemotherapy without sacrificing recurrence-free survival in patients with stage II colorectal cancer (CRC).^1^ This was the first reported prospective, randomized, controlled trial of ctDNA-based interventional adjuvant treatment for stage II CRC.

What can we learn from the DYNAMIC study results? The benefits of adjuvant chemotherapy in stage II CRC remains controversial. Currently, adjuvant therapy is guided by high-risk clinicopathological features. However, the MOSAIC study suggested that high-risk clinicopathological features-guided adjuvant therapy has not been conclusively shown to improve overall survival (OS) in high-risk patients (6-year OS rates: FOLFOX4 vs fluorouracil, 85.0% vs 83.3%, HR, 0.91; 95% confidence interval [CI], 0.61–1.36; *P* = 0.48) and FOLFOX4 did not significantly improve disease-free survival (DFS) compared with fluorouracil arm (82.3% vs 74.6%, HR, 0.72; 95% CI, 0.51–1.02; *P* = 0.063).^2^ Many patients are exposed to unnecessary chemotherapy. Better predictive biomarkers remain to be discovered to help screen patients who can benefit the most from adjuvant treatment.

The DYNAMIC study enrolled patients with resected stage II (T3 or T4, N0, M0) colon or rectal adenocarcinoma. A total of 455 patients were randomly assigned to two arms in a 2:1 ratio: adjuvant therapy guided by ctDNA results (302 patients) or standard clinicopathological features (153 patients). Patients took ctDNA analysis at 4 or 7 weeks after surgery and those who with a positive result were treated with adjuvant chemotherapy. The primary efficacy endpoint was recurrence-free survival (RFS) at 2 years. Compared to the standard treatment group, patients in the ctDNA-guided group had a lower percentage of adjuvant chemotherapy treatment (15% vs. 28%; RR, 1.82; 95% CI, 1.25–2.65). The ctDNA-guided strategy was noninferior to standard treatment for 2-year RFS (93.5% and 92.4%; absolute difference, 1.1%; 95% CI, −4.1–6.2). The RFS rates at 3 years were similar in the ctDNA-guided group and the standard treatment group (91.7% and 92.4%; HR, 0.96; 95% CI, 0.51–1.82).

In recent years, ctDNA has received extensive attention as a promising biomarker. ctDNAs are DNA fragments derived from tumor cells and released into the blood that theoretically have the same genetic and epigenetic characteristics as the cancer cells from which they originated. Studies in recent years have demonstrated that ctDNA analysis can detect the presence of minimal residual disease (MRD) and can be used to predict postoperative tumor recurrence. The CSCO guidelines for CRC version 2022 mentioned that ctDNA monitoring in postoperative patients could be considered.^3^ ctDNA analysis for MRD detection is divided into two methods: tumor-informed analysis and tumor-agnostic analysis. Tumor-informed analysis requires antecedent molecular analysis of the primary tumor which obtained from targeted sequencing or whole-exome sequencing (e.g., Signatera^TM^, SafeSeqS and Droplet digital PCR).^4^ This technique has the advantages of high analytical sensitivity and a low probability of false-positive results, but comes at the cost of longer turnaround times and additional tumor sequencing costs. Conversely, tumor-agnostic analysis, also known as tumor-uninformed analysis, takes advantage of fast turnaround time and conducted to seek genomic alterations and abnormal DNA methylation patterns known to appear in a specific tumor type (e.g., Guardant REVEAL).^4^ Jeanne Tie’s team conducted multiple retrospective analyses of ctDNA detection after surgery and adjuvant therapy for CRC, which confirmed a strong correlation between ctDNA positivity and disease recurrence after surgery and adjuvant therapy. In the ASCO GI 2022, the nonrandomized observational GALAXY study in CIRCULATE-Japan, the largest MRD study to date, confirmed that improved DFS rate was related with ctDNA dynamics in MRD-positive patients. ctDNA clearance rate at 12 weeks and 24 weeks as well as DFS rate at 6 months were significantly higher in adjuvant chemotherapy arm compared to non-adjuvant chemotherapy arm in MRD-positive patients. Ongoing randomized VEGA and ALTAIR studies in CIRCULATE-Japan will further optimize the ctDNA-guided adjuvant strategy.

How long before the ctDNA-guided adjuvant chemotherapy of stage II CRC can reach clinical applications from clinical trials? Two ctDNA tests save nearly half of patients from adjuvant chemotherapy, which is highly encouraging. To answer the question of how long before ctDNA can be routinely used in clinical practice, some pressing issues need to be resolved, including the optimization of detection methods (sensitivity and reproducibility), detection time points and results reporting time, and the ctDNA-guided adjuvant strategies of chemotherapy escalation and de-escalation.

The DYNAMIC study used the tumor-informed personalized ctDNA assays of Safe-Sequencing System. This detection method has high sensitivity and specificity, but the detection steps are complicated, and the detection period is long. In the DYNAMIC study, since ctDNA-positive patients needed to wait for the ctDNA test results, adjuvant chemotherapy was performed 8 to 10 weeks after surgery, which was delayed by about 1 month compared with the standard treatment group (83 days vs. 53 days). This may have affected the efficacy of chemotherapy. In addition, as cell-free DNA from surgical trauma may still influence the ctDNA assay results up to 4 weeks, it was recommended for the assessment of ctDNA status 4–8 weeks after surgical resection.^5^ Therefore, the selection of detection time points and the result reporting time require attention. Before clinical application, standardized ctDNA assay characteristics and the reproducibility of testing results of ctDNA analysis should also be confirmed. Studies have shown that some low-shedding tumors or micrometastases in some specific anatomical sites may cause false-negative ctDNA test results.^6^ Whether patients with undetected preoperative ctDNA should be excluded from ctDNA-guided adjuvant therapy studies, and the additional cost of routine preoperative ctDNA detection, need to be further explored by researchers. The DYNAMIC study has not addressed which adjuvant chemotherapy regimens should be selected based on ctDNA results, and it is unclear whether modifying the start, duration or intensity of adjuvant therapy based on ctDNA results will positively impact patient outcomes. However, some of the ongoing randomized controlled clinical trials related to ctDNA in stage II CRC may give us some insights (Table 1).

**Table 1:** Summary of ongoing clinical trials of ctDNA-guided adjuvant therapy in stage II colorectal cancer (CRC)

Trial Name	Phase	Sample Size	Patient Population	ctDNA assay	Primary Objective
IMPROVE (NCT03637686)	Observational	1800	Stage I–III CRC	Droplet digital PCR	To confirm that ctDNA detected in the plasma after intended curative treatment for CRC can be applied in clinical practice as a marker of subclinical residual disease and risk of recurrence.
IMPROVE-IT2 (NCT04084249)	NA	254	High risk stage II, stage III CRC	Droplet digital PCR (colorectal panel)	To confirm that ctDNA-guided post-operative surveillance combining radiological assessments could detect recurrent disease earlier and screen out more patients eligible for curative treatment.
COBRA (NCT04068103)	Phase 2/3	1408	Stage IIA colon cancer	Guardant LUNAR-1	Phase II: to compare the ctDNA clearance rate in ctDNA-positive patients treated with or without adjuvant chemotherapy after surgery. Phase III: to compare recurrence-free survival (RFS) in ctDNA-positive patients treated with or without adjuvant chemotherapy after surgery.
CIRCULATE (NCT04089631)	Phase 3	4812	Stage II colon cancer (MSS)	Not reported	To compare the DFS in patients who are postoperative ctDNA-positive with and without capecitabine.
CIRCULATE–PRODIGE 70 (NCT04120701)	Phase 3	1980	Stage II colon cancer	ddPCR (2 methylated markers WIF1 and NPY)	To improve the care of patients after surgery, based on ctDNA
CIRCULATE-US (NCT05174169)	Phase 2/3	1912	Stage II–III colon cancer (MSS)	Signatera	To evaluate the kind of chemotherapy to recommend to patients based on the presence or absence of ctDNA after surgery for colon cancer.
CIRCULATE-Japan-VEGA (UMIN000039205)	Phase 3	1240	High-risk stage II, low-risk stage III colon cancer—ctDNA-negative	Signatera	To confirm the non-inferiority between observation vs. adjuvant CAPOX with ctDNA-negative at 1-month post-surgery
CIRCULATE-Japan-ALTAIR (UMIN000039205)	Phase 3	240	Stage II/III CRC or stage IV with resectable metastases	Signatera	To confirm the superiority of trifluridine/tipiracil over placebo in ctDNA-positive patients after standard adjuvant therapy

Increasing the accuracy of recurrence risk prediction after surgery for stage II CRC and the precision of screening patients who can benefit from adjuvant chemotherapy has always been topics that researchers are constantly exploring. Although there are still many questions that need to be verified by more randomized clinical trials, we have to congratulate the DYNAMIC study for its breakthrough and landmark results, which provide high-quality clinical supporting evidence for ctDNA-guided adjuvant chemotherapy strategies. In the future, the results of DYNAMIC study and other ongoing trials may change the level of guidelines recommended for ctDNA testing.
